# Converging phenomics and genomics to study natural variation in plant photosynthetic efficiency

**DOI:** 10.1111/tpj.14190

**Published:** 2019-01-12

**Authors:** Roel F. H. M. van Bezouw, Joost J. B. Keurentjes, Jeremy Harbinson, Mark G. M. Aarts

**Affiliations:** ^1^ Laboratory of Genetics Wageningen University and Research Droevendaalsesteeg 1 6708PB Wageningen The Netherlands; ^2^ Horticulture and Product Physiology Wageningen University and Research Droevendaalsesteeg 1 6708PB Wageningen The Netherlands

**Keywords:** photosynthesis, phenomics, genomics, high‐throughput phenotyping, genome‐wide association study, trait discovery

## Abstract

In recent years developments in plant phenomic approaches and facilities have gradually caught up with genomic approaches. An opportunity lies ahead to dissect complex, quantitative traits when both genotype and phenotype can be assessed at a high level of detail. This is especially true for the study of natural variation in photosynthetic efficiency, for which forward genetics studies have yielded only a little progress in our understanding of the genetic layout of the trait. High‐throughput phenotyping, primarily from chlorophyll fluorescence imaging, should help to dissect the genetics of photosynthesis at the different levels of both plant physiology and development. Specific emphasis should be directed towards understanding the acclimation of the photosynthetic machinery in fluctuating environments, which may be crucial for the identification of genetic variation for relevant traits in food crops. Facilities should preferably be designed to accommodate phenotyping of photosynthesis‐related traits in such environments. The use of forward genetics to study the genetic architecture of photosynthesis is likely to lead to the discovery of novel traits and/or genes that may be targeted in breeding or bio‐engineering approaches to improve crop photosynthetic efficiency. In the near future, big data approaches will play a pivotal role in data processing and streamlining the phenotype‐to‐gene identification pipeline.

## Significance Statement

The fields of genomics and phenomics in plant science have gone through a major revolution that has led to the technological and methodological maturation of both fields of study in the past decade. Expansion of high‐throughput phenotyping technology will soon allow the scaling up of forward genetics approaches in order to adequately study the genetic architecture of traits that may accelerate the much needed improvement of photosynthesis in crop plants.

**Glossary**

*Photosynthesis‐related trait*: In this review we consider photosynthesis‐related traits to be those that directly affect photosynthetic functioning (e.g. chlorophyll content, photosynthetic efficiency, non‐photochemical quenching). The exact boundary of what constitutes the photosynthesis‐related trait, however, remains debatable.

*Forward genetics*: All methodology (of which genetic mapping is the most common) in which functional genetic variation is derived from a phenotype using populations containing genetic variation. Reverse genetics approaches assess the effect of known or artificially induced genetic mutations or polymorphisms on a phenotype.

*Diversity panel*: A collection of genetic variants. In genome‐wide association mapping, panels are collected from naturally occurring populations of wild plant species, or wide germplasm collections of crop species, with the plan that they represent a large fraction of the existing globally or geographically specific genetic variation of a species. This in contrast to artificial, experimental populations in which genetic variation of a limited number of founder lines is allowed to segregate in recombinant offspring.

*Quantitative trait locus (QTL)*: In genetic mapping, a QTL represents a genomic region that is genetically linked to functional genomic variation and significantly associated with quantitative phenotypic trait variation.

*Logarithm of the odds (LOD) score*: A measure of the statistical likelihood of the presence of a QTL, typically calculated as the –log(*P*‐value) from the analysis of variance, by using a bi‐allelic polymorphism as a factor. Prior to QTL analysis, a LOD threshold significance is set to determine the confidence level of detected QTLs.

## Introduction

In the past two decades annual increases in yield of major staple food crops such as rice, wheat, soybean and maize have stagnated globally despite agronomic and genetic improvements (Wei *et al*., 2015). It is expected that by the middle of this century 60–110% greater yield output is required from crops for food, feed and bio‐fuel in order to keep up with the increasing demand of the growing human population worldwide (Kromdijk and Long, 2016; Tilman *et al*., 2011). To keep pace with this development a new Green Revolution will be required to double the realised average annual yield increment from 2000 until 2018 of approximately 1.2%, up to a minimum of 2.4% per year from 2018 until 2050 (Ray *et al*., 2013). The forecast negative effects of global warming on the yield of staple crops will further complicate the achievement of this goal, given that the negative effects largely offset the potential gains from increased atmospheric carbon dioxide (Schauberger *et al*., 2017; Zhao *et al*., 2017). The relationship between irradiance and crop yield is summarised by the yield model of Monteith (Long *et al*., 2006) in which the harvestable yield component is the product of irradiance, the interception of irradiance, the efficiency of conversion of intercepted irradiance into biomass (ε_c_) and the harvest index. In the past, crop breeding programmes aimed to increase the harvest index and the light capture efficiency of the canopy, and did so successfully. As a result these traits are now reaching their theoretical potential, making it unlikely that there can be further significant improvement (Long *et al*., 2006; Zhu *et al*., 2010). The remaining component of yield, ε_c_, is strongly influenced by the efficiency of photosynthesis, and ε_c_ is still far below its the theoretical maximum (Zhu *et al*., 2010). This means that there is still room for improving this trait by crop breeding. A modest 50% improvement in ε_c_ – which would still leave it far below its theoretical maximum – would already be enough to bridge the gap between human demand and production of plant resources. Given its importance to ε_c_, photosynthesis is considered as a primary target for improvement in crop species (Long *et al*., 2006; Ort *et al*., 2015; Long *et al*., 2015).

The physiological basis of photosynthesis, and the genes coding for the proteome of photosynthesis, have been relatively well characterised during the last decades (e.g. Farquhar *et al*., 1980). In the past 15 years, attempts to improve plant photosynthetic efficiency have focused on identifying the efficiency bottlenecks in photosynthesis and then bio‐engineering photosynthetic pathways to overcome them (Long *et al*., 2015). This approach has led to suggestions, amongst others, to modify specific subunits of photosystem II (PSII) to optimise light‐harvesting capacity (Long *et al*., 2006; Ort *et al*., 2011; Walker *et al*., 2018), improve the catalytic properties of Rubisco (Carmo‐Silva *et al*., 2014) and transplant the C4 photosynthesis machinery into C3 plants (Crovshoff and Hibberd, 2012; Schüler *et al*., 2016). Application of this strategy has been successful. For example, increasing the speed with which the photosynthetic machinery in tobacco adapts to fluctuating light resulted in greatly improved average photosynthetic efficiency throughout the photoperiod (Kromdijk *et al*., 2016). Improving the rate of ribulose‐1,5‐bisphosphate (RuBP) regeneration in the Calvin cycle in wheat by over‐expressing sedoheptulose‐1,7‐bisphosphatase (SBPase) resulted in increased rates of CO_2_ fixation (Driever *et al*., 2017). These improvements in photosynthesis translate to increases in plant biomass: in the case of tobacco, with improved responses to fluctuating light (Kromdijk *et al*., 2016), plant biomass was increased by 14–20% under field conditions, while for wheat, with improved RuBP regeneration, a 30–40% higher grain yield was obtained under greenhouse conditions (Driever *et al*., 2017). From these examples it should be clear that improving photosynthetic efficiency is both a promising and feasible route for increasing crop yields.

Natural variation is an underexploited genetic resource with which to improve plant photosynthesis and the genetic yield potential of crops (Flood *et al*., 2011; Lawson *et al*., 2012). The failure to better use natural variation in photosynthesis as a route to yield improvement stems partly from the misconception that photosynthesis has been fully optimised by millions of years of selection for this vital trait (Flood *et al*., 2011). Clearly this assumption of perfection is misguided – if nothing else, the improvements in photosynthesis outlined above show this. In addition, highly heritable variation in photosynthesis‐related traits has been found for model and crop species such as wheat (Driever *et al*., 2014), Arabidopsis (*Arabidopsis thaliana*), soybean (*Glycine max*), sorghum (*Sorghum bicolor*), maize (*Zea mays*) and rice (*Oryza sativa*) (see Table 1). The identification of functional allelic variations of important genes and traits related to photosynthesis using a forward genetics analysis of diversity panels is therefore a valuable approach, for three main reasons. First, the study of natural variation offers key insights into regulatory processes of photosynthesis, for example under fluctuating light conditions (Lawson *et al*., 2012; Murchie *et al*., 2018). Furthermore, genetic variation in the core genes of photosynthesis has been acknowledged to be an important resource for the bio‐engineering of improved photosynthesis (Prins *et al*., 2016; Reeves *et al*., 2018). Lastly, when beneficial alleles are identified they can be exploited in current crop breeding programmes. The exploitation of natural variation in photosynthesis is likely to be particularly active in jurisdictions where the use of genetically modified crops is prohibited, or where the resources to generate genetically modified crops are not available. Note, however, that genes identified as being important in determining photosynthetic traits based on studies of natural variation of these traits could be valuable not only in conventional plant breeding approaches to improving photosynthesis but also in genetic modification approaches.

**Table 1:** An overview of genome‐wide association analyses found in the literature that target photosynthesis‐related traits

Species	Nature of data (photosynthesis‐related only)	Stress	Data	Growth conditions	Rep	Traits (photosynthesis‐related only)	Study
Soybean	Single measurements of chlorophyll content by extracts and spectral measurements	–	1	Climate chamber	1	Chlorophyll content and chlorophyll reflectance traits	Hao *et al*. (2012)
Maize	Single measurements of data using handheld pulse amplitude modulation (PAM) and soil plant analysis development (SPAD) index devices	Cold	1	Climate chamber and field grown	1	Chlorophyll content (SPAD) and chlorophyll reflectance traits	Strigens *et al*. (2013)
Rice	Single measurements of chlorophyll content by both leaf extracts and spectral measurements in mature plants	–	1	Field grown	2	Chlorophyll content (SPAD and leaf extracts)	Wang *et al*. (2015)^b^
Arabidopsis	Photosystem II efficiency data taken 1 h after a stepwise increase from low to high light	High light	1	Climate chamber^b^	1	Photosystem II (PSII) efficiency (PAM derived)	van Rooijen *et al*. (2015)^II^
Soybean	Single measurement of chlorophyll reflectance index of soybean canopies using multispectral devices	–	1	Field grown	2	Non‐photochemical quenching (NPQ) (derived by broadview multispectral devices)	Herritt *et al*. (2016)^a^
Soybean	Measurements derived from handheld PAM‐devices and assessment of chlorophyll content in seedling plants at multiple levels of cold stress	Cold	1	Climate chamber	1	Chlorophyll content (SPAD) and chlorophyll reflectance traits	Fiedler *et al*. (2016)
Soybean	Single measurement of chlorophyll content by both extracts and spectral measurements	–	1	Field‐grown	2	Chlorophyll content (multispectral derived and leaf extracts)	Dhanapal *et al*. (2016)^a^
Rice	Measurements of NPQ in excised leaf discs by handheld PAM devices	–	1	Field grown	2	NPQ (PAM derived)	Wang *et al*. (2017)^b^
Sorghum	Pre‐cold, cold and post‐cold measurements of chlorophyll fluorescence traits and gas‐exchange measurements	Cold	3^a^	Climate chamber	1	Chlorophyll reflectance and gas‐exchange traits	Ortiz *et al*. (2017)
Arabidopsis	PSII efficiency measurements, under control (2 days) and high light (4 days) conditions	High light	18	Climate chamber^b^	1	PSII efficiency (PAM derived)	van Rooijen *et al*. (2017)^II^
Rice	Three measurements of chlorophyll content by SPAD meter in developing seedlings	–	3	Climate chamber	1	Chlorophyll content (SPAD)	Lin *et al*. (2017)

a Before, during and after cold measurements.

b See Flood *et al*. (2016bb) for a description.

Nature of data: brief description of the methodology of obtaining the data. Stress: Stresses subjected to plants as given in the corresponding literature. Growth conditions: Growth conditions of the experiment. Rep: describes the number of replicates of the entire experiment to obtain the complete dataset. Traits: the type of traits that were analysed in the study. Study: Roman numerals I, II and III correspond to repeated use of data collection obtained from the same experiment as described in the earliest published papers’ methods of each pair.

**Figure 1:**
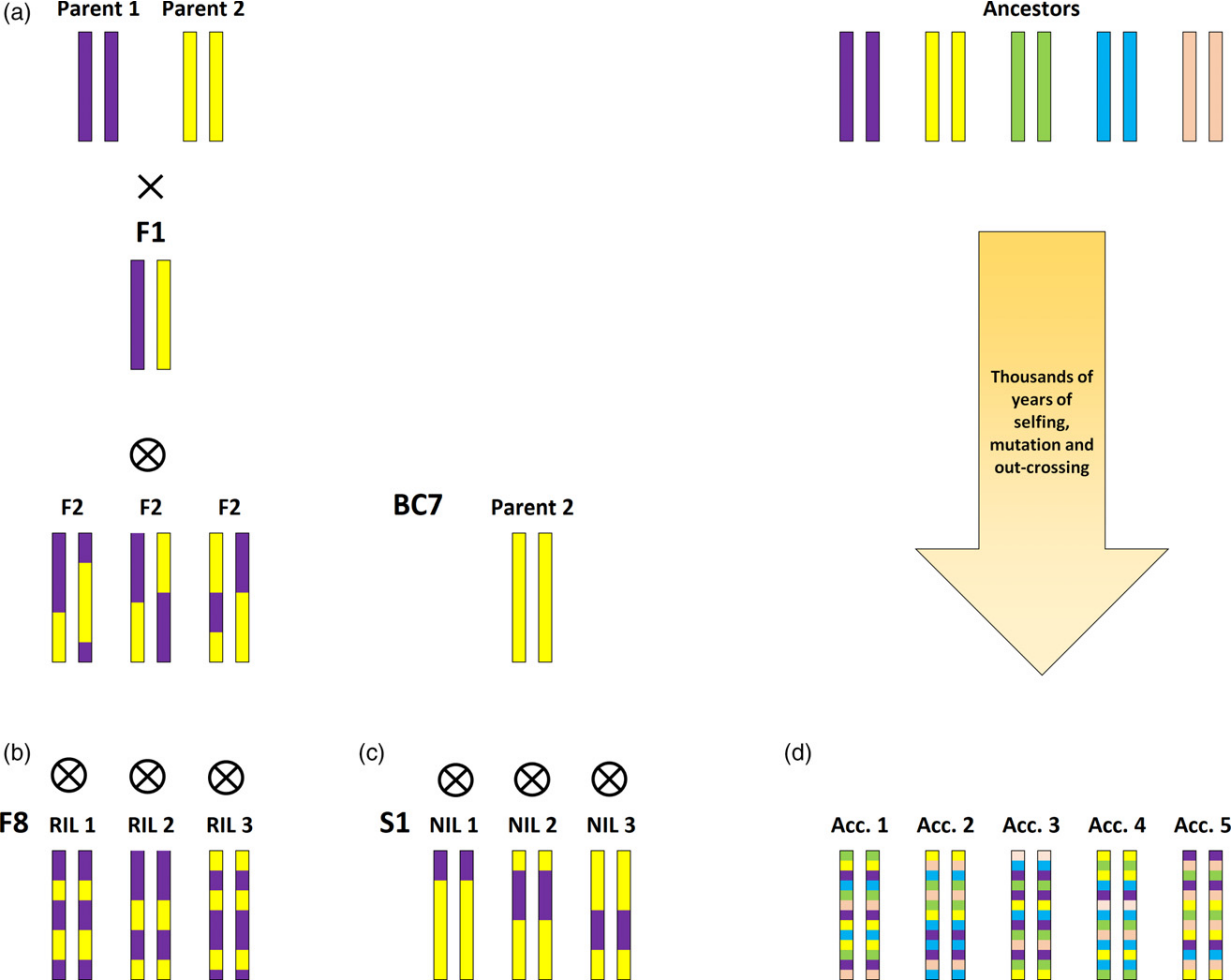
Schematic overview of the generation of different genetic mapping populations in inbreeding plant species. (a) F_2_ populations derived from a self‐fertilised F_1_ require only two generations to produce. (b) Further self‐fertilizing of F_2_ lines for more than eight generations through single‐seed descent will result in fully homozygous recombinant inbred lines (RILs). (c) Parental backcrossing of the F_2_ while selecting for specific chromosomal segments will result in homozygous near isogenic lines (NILs) after more than seven generations and a single round of self‐fertilizing. (d) Diversity panels are collections of independent, self‐fertilised accessions (Acc.) that result from thousands of years of self‐ and cross‐fertilization and mutating between ancestral genotypes that can no longer be retraced.

**Figure 2:**
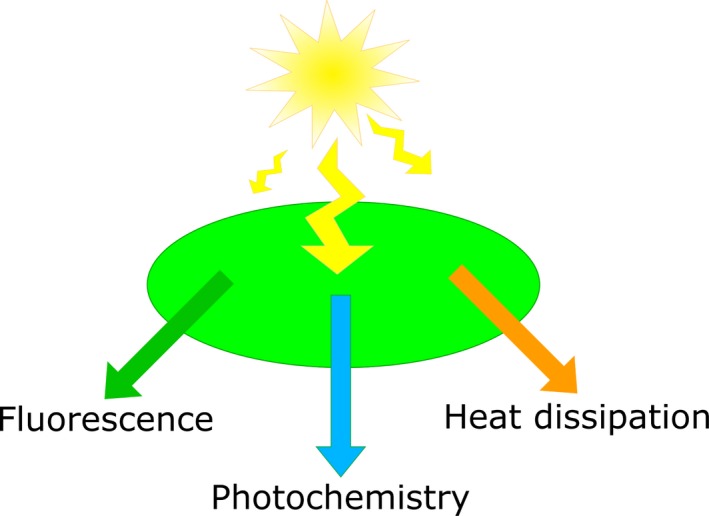
The fate of quanta absorbed by photosystem II. The absorption of photons by photosystem II results in the formation of excited, energy‐rich chlorophyll *a* molecules. These can be used to drive photochemistry, they may lose their energy as heat or they may lose the energy by re‐emitting it as a photon (fluorescence). Photochemistry in photosystem II drives the oxidation of water and the reduction of plastoquinone and is the start of the linear electron transport chain. The linear electron transport chain drives carbon assimilation. Heat dissipation in the form of non‐photochemical quenching is a regulated process that is controlled to dissipate excited states of chlorophyll *a* that are in excess of the needs of photochemistry. Non‐photochemical quenching is a protective process, as by dissipating the excited states as heat it reduces the potential for producing damaging reactive oxygen species in photosystem II. Whenever the rate of one of these parameters changes, the rates of the other two are adjusted proportionately. This makes chlorophyll fluorescence a sensitive and thereby extremely useful tool for assessing photosynthetic performance in plants.

Despite the potential for increasing photosynthetic efficiency, major bottlenecks in the phenotypic and genotypic evaluation of photosynthesis‐related traits exist (Flood *et al*., 2011; Murchie *et al*., 2018). In the last 10 years, however, both plant genomics and phenomics have matured to the point where, together, they can be used in forward genetics analyses of photosynthetic traits. In the case of photosynthesis, chlorophyll fluorescence techniques allow the measurement of a range of photosynthetic traits (Baker, 2008; Maxwell and Johnson, 2000; Murchie and Lawson, 2013) and fluorescence imaging techniques allow these traits to be measured quickly for large numbers of plants, enabling the high‐throughput phenotyping of photosynthesis (Box 1, Figure 1). At the same time, whole‐genome sequencing has become cheaper (Barabaschi *et al*., 2016; Jiao and Schneeberger, 2017), leading to the development of high‐resolution mapping populations in the form of genotypically detailed diversity panels suitable for genome‐wide association studies (GWAS) (Box 2, Figure 2). This convergence of phenomics and genomics is expected to lead to a better understanding of the genetics and molecular mechanisms of complex traits such as photosynthesis. Nonetheless, large‐scale and detailed phenotyping of photosynthetic traits is relatively new and may be conceptually challenging due to the multidimensional nature of the traits. The possibilities for high‐throughput phenotyping of many plant traits have been reviewed in detail in other works (e.g. Awlia *et al*., 2016; Rungrat *et al*., 2016; Großkinsky *et al*., 2017). If measurement of photosynthesis‐related traits is discussed it is usually limited to the context of abiotic stress responses (Rungrat *et al*., 2016, Großkinsky *et al*., 2017). We believe that the time has come to examine the plethora of genomic and phenomic tools that have been developed and how these can be applied to successfully screen for natural variation in photosynthesis beyond its relatively simple use as a measure of plant stress.

Box 1 **Genetic mapping populations for trait discovery**

Quantitative trait locus (QTL) mapping is traditionally performed by crossing two distinct genotypes with contrasting phenotypes (parents) to generate an F_1_ generation and then subsequently subjecting the F_2_ or other next‐generation progeny to phenotypic screening. By extensive genotyping of the F_2_ generation using genomic markers [often based on single nucleotide polymorphisms (SNPs) or insertion–deletion (InDel) polymorphisms in the genomes of two parental lines], causal loci responsible for the observed differences in phenotype can be identified in QTL analysis, and will thus provide insights into the genetic architecture of the studied trait. Each genotypic marker is assessed by applying analysis of variance (ANOVA), and when the significance reaches a pre‐formulated logarithm of the odds (LOD) threshold the genomic region is claimed to be a QTL for a phenotype.

Major disadvantages of mapping in an F_2_ population are that there are no replicates for each genotype and that heterozygosity is prevalent in such a population, causing further segregation in subsequent generations (Figure 1a) – both of which will increase genotypic variance that is difficult to properly account for in QTL mapping. In addition, the limited number of recombination events per chromosome generated by a single round of self‐fertilisation means that genetic linkage between distant regions is strong and large populations will need to be screened to achieve a sufficient mapping resolution to be able to pinpoint genomic regions of interest.

In order to overcome most of the disadvantages described above, so‐called ‘immortal’ inbreeding and backcrossing populations have been developed in plant species that allow inbreeding (Wijnen and Keurentjes, 2014). The most common ones are recombinant inbred line (RIL) and near isogenic line (NIL) populations (Bazakos *et al*., 2017; Keurentjes *et al*., 2007). Recombinant inbred line populations are acquired by selecting several F_2_ plants and repeatedly self‐pollinating these plants for six to eight generations (Figure 1b). This will result in near homozygosity of all alleles and a large number of recombination events which will increase mapping resolution. Near isogenic line populations are generated through repeated backcrossing of F_2_ plants to a recurrent parental line (Figure 1c). Molecular markers are then used to identify genomic regions of interest which will be selected for until the genetic background is isogenic to that of the parent. Near isogenic line populations effectively allow the study of genomic regions in isolation and individual lines can be easily backcrossed again to the parent of interest to fine map causal loci. The genetic layout of each of these solutions introduces different types of population structure and experimental pre‐conditions that should be taken into account to properly identify genes related to traits (Keurentjes *et al*., 2007).

The ever decreasing costs of full genome sequencing (Barabaschi *et al*., 2016) have led to the development of diversity panels suitable for GWAS in many crop and model species (Bazakos *et al*., 2017; Ingvarsson and Street, 2011; Korte and Farlow, 2013). These studies exploit naturally occurring variants resulting from bi‐allelic SNPs that have been recombining between genotypes through the course of history of plant species (Figure 1d), as identified through whole genome sequencing. In this way, thousands to millions of SNPs can be identified in diversity panels, to full saturation of the genome. The process of continuous recombination out‐crossing events typically lasts thousands to millions of years in such panels. Co‐segregating small genomic regions (also called linkage or haplotype blocks) in wild species often span less than 1 kilobase pair (kbp). In crop species that have only recently been domesticated, the size is generally less than 100 kbp. Compared with the linkage blocks of a few million base pairs that are typically found in bi‐parental mapping populations such as F_2_, RILs and NILs, this is a huge improvement. The result is that once a genomic region has been identified through QTL mapping there will be only a few relevant candidate genes. This strongly accelerates gene identification and overcomes the requirement for additional fine mapping experiments, especially for highly polygenic traits such as photosynthesis; this has led to GWAS being widely adopted by forward plant geneticists (Atwell *et al*., 2010; Ingvarsson and Street, 2011; Tian *et al*., 2011).

Potential disadvantages of GWAS, compared with conventional bi‐parental mapping studies, generally include the low statistical power of finding QTLs. One of the reasons for this is that many independent marker–trait association tests need to be made – which increases the threshold to control for false positives. Additionally, in an ideal RIL population all alleles are present in a 1:1 ratio, while allelic distributions in GWAS populations may be heavily skewed towards the major allele (Figure 1d), further compromising QTL detection power (Korte and Farlow, 2013). Furthermore, the two‐dimensional nature of bi‐allelic SNPs may be an oversimplification of reality since in natural populations many different functional alleles exist and introduce extra variance into the analysis (Forsberg *et al*., 2015; Ingvarsson and Street, 2011; Korte and Farlow, 2013). On top of these potential disadvantages, epitasis and epigenetics may further introduce variance that is difficult to account for in statistical models. Nevertheless, GWAS in inbreeding plant species can be considered highly potent since replications can be used, which opens the possibility of repeating the same experiment for confirmation or to account for genotype–environment interactions (Brachi *et al*., 2011).

Box 2 **Evolution of photosynthesis phenotyping tools and their potential for phenomics**

The genetic mapping of photosynthesis‐related traits requires robust and efficient phenotyping protocols that can be used to reliably assess these traits between different plant genotypes. A range of methods have been developed in order to estimate the performance of photosynthesis‐related traits in plant leaves. The most important ones are listed here.

**Measurement of leaf gas exchange**

Infrared gas analysis technology allows direct measurements of carbon dioxide uptake and release and water vapour release by plants (Long *et al*., 1996). Infrared gas‐analyser systems intended for the measurement of photosynthetic gas exchange are available as highly integrated systems, including, for example, measurement systems for leaf temperature, irradiance and chlorophyll fluorescence, and with extensive on‐board data processing. Infrared gas analysis systems are accurate, but also expensive, and the measurement protocols are generally time‐consuming (Stinziano *et al*., 2017). They require an operator, making them unsuitable for robotic use. Recently a method for the faster measurement of *A*/*Ci* curves (net CO_2_ assimilation rate, *A*, versus calculated substomatal CO_2_ concentration, *Ci*) has been proposed, allowing a complete *A*/*Ci* curve to be measured in about 5 min (Stinziano *et al*., 2017) (this corresponds to about 100 assessments per day). While this is a welcome advance, it needs to be seen in the context of the potential need to measure 1000–2000 plants for a genome‐wide association analysis, and to do this quickly enough to avoid problems due to the variation in assimilation that can occur over even a day.

**Assessment of chlorophyll content and other leaf reflectance‐based methods**

The reflectance spectrum of a leaf and changes in this reflectance spectrum can be a useful source of biochemical and physiological information. In some cases reflectance‐based measurements have been developed for high‐throughput phenotyping applications (e.g. measurement of the content of leaf chlorophyll and other foliar pigments). In other cases optical methods, which have been developed for more intensive laboratory applications, have not yet been used for high‐throughput applications despite being, in principle, suitable for this.

Measurement of chlorophyll content is often performed using laborious destructive protocols, which greatly reduce their suitability for the high‐throughput assessment required in genetic mapping studies. Another problem arises from the variation of chlorophyll content over time (Flood *et al*., 2016b; Hardwick and Baker, 1974; Lin *et al*., 2017). The foliar content of chlorophyll and other photosynthetic (and non‐photosynthetic) pigments can be measured via leaf reflectance measurements (Gitelson *et al*., 2001, 2003, 2006) or by means of chlorophyll fluorescence (Cerovic *et al*., 2002; Gitelson *et al*., 1999). These essentially optical methods can be readily implemented using narrow‐band (i.e. via optical filters; e.g. Flood *et al*., 2016b) or (hyper)spectral imaging (Yendrek *et al*., 2017), making them suitable for high‐throughput phenotyping.

Leaf reflectance or absorbance measurements have also been used to estimate physiological properties connected with photosynthesis. The photochemical reflectance index (Gamon *et al*., 1997), which is correlated with the de‐epoxidation state of the xanthophyll‐cycle pigment pool and thus the qE component of non‐photochemical quenching (NPQ) (Gamon *et al*., 1997; Herritt *et al*., 2016; Ruban *et al*., 1993), has been developed for field‐ and even satellite‐based remote sensing applications (Drolet *et al*., 2005). The physiological traits *Vc*_max_ and *J*_max_, which are commonly obtained from an analysis of *A*/*Ci* curves, have been estimated from near‐infrared reflectance data (Silva‐Perez *et al*., 2018) using an approach that would be highly suited to high‐throughput applications. Other light‐induced absorbance change techniques that have been developed for intensive measurements of photosynthesis (the electrochromic shift and absorbance changes induced by near‐infrared light; Baker *et al*., 2007) could be used in imaging‐based, high‐throughput applications – if this is technically feasible. So far, however, there are no examples of these other potentially useful techniques being applied in fully automated, high‐throughput applications, although some have been refined for use in hand‐held field phenotyping instrumentation (Kuhlgert *et al*., 2016).

**Chlorophyll fluorescence‐based techniques**

Chlorophyll fluorescence has been used extensively to measure photosynthetic processes in chlorophyll‐containing samples, such as isolated photosynthetic pigment‐binding complexes, isolated thylakoids, chloroplasts and leaves (reviewed in depth by Baker, 2008; Harbinson, 2018). Chlorophyll fluorescence is the reverse of the light absorption process that forms excited states of chlorophyll *a* in the first place, and while fluorescence occurs from all chlorophyll, it is from PSII that most fluorescence is emitted and the yield of PSII fluorescence is strongly influenced by its physiological state (Figure 2). The most important traits derived from chlorophyll fluorescence are photosynthetic efficiency (*F*_q_′/*F*_m_′), which is a measure of photosynthetic efficiency in light‐adapted leaves, and the maximum photosynthetic efficiency (*F*_v_/*F*_m_) measured in dark‐adapted leaves under conditions where the yield of photochemistry is maximal and no rapidly relaxing NPQ is present. Modulated chlorophyll fluorescence techniques are the most commonly used methods for measuring chlorophyll fluorescence *in folio*, and with these techniques a wide range of physiologically useful parameters can be derived (Baker, 2008; Harbinson, 2018; Murchie and Harbinson, 2014). Chlorophyll fluorescence‐based techniques are continuously being developed in order to improve their efficiency. For example, a single assessment of NPQ in plants requires a long dark‐adapted phase lasting for 10–20 min (Baker, 2008; Murchie and Lawson, 2013). To overcome this requirement, Tietz *et al*. (2017) developed a novel protocol termed NPQ_(T),_ which drastically improved the speed at which each measurement takes place. The relative ease with which chlorophyll fluorescence methods can be applied using imaging techniques means that they are widely used in the high‐throughput phenotyping of photosynthesis.

This review aims to bring together developments in genomics and phenomics from the past 10 years, and evaluate their progress within the context of exploring natural variation in photosynthesis. We first aim to provide an overview of how insights into photosynthesis and genetic mapping studies have contributed to a deeper understanding of the genetic architecture of photosynthesis. As such, we have reviewed studies that aimed to unravel the genetics of photosynthesis by using phenotyping methods that are directly linked to photosynthesis‐related traits and omit studies that assessed indirect traits, such leaf architecture. Next, phenotyping of photosynthesis‐related traits will be critically reviewed in the context of mapping trait variation. In addition, we will argue for the need for phenomics to overcome the challenges that lie ahead of successful mapping. Finally, high‐throughput phenotyping of photosynthesis in relation to statistics, data handling and gene identification will be discussed.

## Basic Principles of the Green Machine

**Figure 3:**
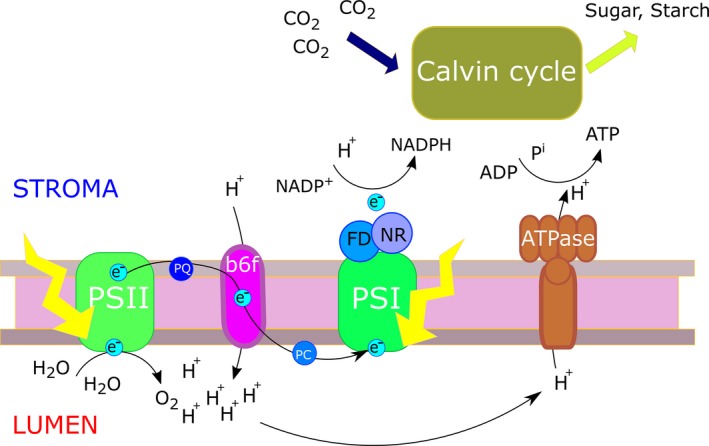
A summary of photosynthesis. Light energy is used by photosystem II (PSII) to oxidise water and reduce plastoquinone (PQ) to plastoquinol. The reducing equivalents on plastoquinol move through the electron transport chain via the cytochrome *b*_6_/*f* complex and plastocyanin, and are used by photosystem I (PSI) to reduce ferredoxin, a process that is also light‐driven. Reduced ferredoxin in turn reduces NADP ^+^ to NADPH via the enzyme ferredoxin NADP oxidoreductase (NR). The oxidation of water by PSII and the oxidation of plastoquinol by the cytochrome *b*_6_/*f* complex liberates protons (H^+^) into the thylakoid lumen, generating a proton potential difference between the thylakoid lumen and the stroma. Protons passing down this proton potential difference through the ATPase drive the phosphorylation of ADP to form ATP.

Photosynthesis is a diverse physiological process that couples the energy of absorbed quanta to metabolism, permitting otherwise endergonic reactions to proceed. Oxygenic photosynthesis, which occurs in cyanobacteria, algae and plants, is the dominant type of photosynthesis and sustains most life in the biosphere. In short, photosynthesis comprises the following steps: (i) light absorption by photosynthetically coupled pigments (primarily chlorophylls) in PSI and PSII; (ii) the use of this absorbed energy to drive chemical reactions that result in the formation of energy‐rich metabolically useful compounds (reduced ferredoxin or NADPH, and ATP); (iii) and metabolic activity driven by NADPH, ferredoxin or ATP (see Figure 3 for an overview). The most conspicuous of the metabolic processes coupled to photosynthesis is CO_2_ fixation, which is driven by the light‐independent photosynthetic carbon reduction cycle (also called the Calvin cycle), which eventually results in the formation of sugars (initially as sugar phosphates) and starch. Next to underpinning most life in the biosphere, photosynthesis is the fundamental process for agricultural productivity. Photosynthetic efficiency, i.e. the efficiency with which light is used to drive the photosynthetic processes, is an essential measure of photosynthetic performance (Baker, 2008; Maxwell and Johnson, 2000). It effectively dictates the amount of light energy that is required in photochemistry to fix a unit of carbon dioxide. It is this process of carbon dioxide fixation that contributes most to biomass formation. The regulation of photosynthetic light harvesting and electron transport is dominated by the need to moderate the production of reactive oxygen species (ROS) as a by‐product of photosynthetic chemistry. Both superoxide and singlet dioxygen (molecular oxygen) can be formed directly by the photosynthetic light‐harvesting and electron transport systems. To this end, various processes, such as NPQ, the production of anti‐oxidants and the regulation of electron transport, are present to moderate the formation of ROS and detoxify them if they are formed. These protective mechanisms are activated in response to stressful light conditions, either due to too much incoming light and/or as a result of physiological stress, to reduce the rate of formation of ROS by photosynthesis.

Non‐photochemical quenching in PSII is an important photoprotection mechanism. The absorption of quanta by PSII results in the formation of excited states of chlorophyll *a* in PSII. These are used to drive photochemistry in PSII and photosynthetic electron transport that result in the formation of NADPH and ATP. If the rate of formation of excited states of chlorophyll *a* in PSII exceeds the metabolic demands of photosynthetic metabolism, and thus photochemistry in PSII, the activation of NPQ ensures that the excess of excited states is dissipated as heat (Murchie and Harbinson, 2014). The activation of NPQ depends on decreases in the pH of the thylakoid lumen which will occur if the rate of proton deposition into the lumen, as a result of photosynthetic electron transport, exceeds the use of these protons by ATP synthesis (Figure 3). An increase in proton concentration leads to a fall in lumen pH, protonation of photosystem subunit S (PsbS) and the activation of violaxanthin epoxidase, which leads to the epoxidation of violaxanthin to antheraxanthin and eventually zeaxanthin (Latowski, *et al.,* 2011). Both the protonation of PsbS and the formation of antheraxanthin and zeaxanthin contribute to the formation of NPQ in PSII. Essentially then, NPQ thermally dissipates excited states of chlorophyll *a*, reducing the concentration of these excited states in PSII and reducing the risk of photodamage due to ROS formation. When light conditions become favourable again (i.e. lower), or when the plant adapts to the new light environment (i.e. photosynthetic capacity increases), lumen pH increases, deprotonating PsbS and activating a de‐epoxidase that converts zeaxanthin and antheraxanthin back to violaxanthin. In this way, NPQ protects the integrity of the photosystems and reduces photodamage (Müller *et al*., 2001).

## The Genomic Complexity of Photosynthesis

Photosynthesis is arguably the trait that above all others sets plants and algae apart from other, heterotrophic, eukaryotes, and their needs have largely driven the evolution of plants as terrestrial organisms over the past 450 million years. The importance of photosynthesis to the nature of plants can be seen by comparing parasitic plants with their more typical autotrophic relatives. The anatomy of parasitic plants is often reduced to only haustoria‐like structures and reproductive organs (Kokla and Melnyk, 2018), and frequently there is the loss of plastids and photosynthetic genes (Hadariová *et al*., 2018; Vogel *et al*., 2018).

In the course of evolution, many genes originally residing on the chloroplast genome have moved to the nuclear genome, but their proteins are still mostly active in chloroplasts. A specific, conserved, sequence tag encoding the so‐called ‘chloroplast transit peptide’ needs to be present in genes located on the nuclear genome in order for the translated protein to be properly transported into the chloroplasts (Bruce, 2000). Using the presence of this sequence tag for the transit peptide as a criterion for a photosynthetic gene, roughly 10–15% of plant genes are involved in photosynthesis (AGI 2000, Leister, 2003; Richy and Leister, 2004; van Rooijen *et al*., 2015). Given that the chloroplast genomes of embryophytes are estimated to contain only about a hundred of these genes, most of the genes coding for chloroplast‐targeted proteins reside within the nuclear genome and can be genetically mapped – which amounts to roughly 3000 genes in the genome of Arabidopsis alone.

Although mutations in the chloroplast genomes can potentially have strong adverse effects on photosynthesis in plants they cannot be genetically mapped due to their strictly maternal inheritance in nearly all species and the absence of meiotic recombination. For example, a single nucleotide mutation in the chloroplast gene *Photosynthesis Subunit A* (*PsbA*) reduces the photosynthetic efficiency of Arabidopsis (El‐Lithy *et al*., 2005)*. PsbA* encodes for the D1 protein, a reaction centre protein of PSII, so it is involved in charge separation in PSII and the subsequent transfer of electrons to downstream electron acceptors (Figure 3). This process is blocked entirely by binding of the herbicide atrazine to the D1 protein (Barros and Dyer, 1988; Kuhn and Böger, 1990). A point mutation causing a Ser264–Gly substitution in D1 leads to a reduction in the efficiency of PSII charge separation (Barros and Dyer, 1988; Kuhn and Böger, 1990). This mutation prevents atrazine from binding to the D1 protein, making the plant atrazine resistant, but at the cost of reduced photosynthetic efficiency and reduced growth. Despite the reduction in growth this is a viable phenotype, especially in atrazine‐treated areas (El‐Lithy *et al*., 2005; Flood *et al*., 2016a). This shows that strong artificial selection pressure is required in order to select for substantial negative chloroplast effects on photosynthetic efficiency. The contribution of other functional chloroplastic (or even mitochondrial) variation to phenotypic variance for photosynthesis in natural genetic populations or crop species accessions is currently unknown. However, such variation may in part be responsible for genetic variance for photosynthesis‐related traits that cannot be accounted for in GWAS, also known as missing heritability (Brachi *et al*., 2011; Zuk *et al*., 2012). Analytical pipelines or statistical models that can take this variation into account will need to be developed. Identification of the phenotypic effect of chloroplast mutations could be performed by comparison of reciprocal F_2_ or RIL mapping populations, in which the nuclear genome segregates in two different cytoplasmic backgrounds (El‐Lithy *et al*., 2005). Alternatively, cybrids, cyto‐swaps or cytolines may be used, in which a full nuclear genome is transferred to another cytoplasmic background, replacing the original nuclear genome, for example by recurrent backcrossing (Miclaus *et al*., 2016; Roux *et al*., 2016).

The conserved composition of photosynthetic complexes coupled with the importance of the process implies that not all nuclear‐encoded chloroplast‐targeted proteins are likely to have significant natural genetic variation (e.g. essential genes involved in maintaining the chloroplast membranes). For other genes associated with photosynthesis, but not coding for elements of the typically multicomponent photosynthetic complexes and enzymes, the situation may be different. van Rooijen *et al*. (2015) identified SNPs associated with variation in the recovery of photosynthetic efficiency after transition from low to high light. Associated with these SNPs were genes so far not linked to photosynthesis, although these were enriched for genes encoding chloroplast proteins. The large number of genes encoding chloroplast‐targeted proteins found in the nuclear genome still leaves plenty of room for the discovery through forward genetics of novel nuclear genes involved in photosynthesis‐related traits. Sequencing efforts in parasitic plants, which have lost a significant part of their photosynthetic capability, may provide further insight into their identification (Vogel *et al*., 2018). The fact that little research has been performed to identify such genes means that there is also still a lot to discover about the function of such novel genes in photosynthesis.

## Insights from Genetic Mapping Studies into the Genetic Architecture of Plant Photosynthesis

From the above it is clear that photosynthesis is a complex quantitative trait that involves many genes. Quantitative trait locus mapping is an effective and widely used research tool to unravel quantitative traits, as it allows the unbiased discovery of the underlying functional variants of relevant genes. The diversity of genetic mapping studies seeking to dissect the genetic architecture of photosynthesis‐related traits has increased markedly, which can be attributed to the awareness of the need to improve photosynthesis (Flood *et al*., 2011; Leister, 2003; Long *et al*., 2006; Zhu *et al*., 2010). Popular QTL‐mapping population types such as RIL and NIL populations have been used to discover genetic variation and genes that contribute to variation in photosynthetic efficiency for a wide variety of species (e.g. Adachi *et al*., 2011, 2014; Gu *et al*., 2012; Yan *et al.,* 2015; Oakley *et al*., 2018; de Oliveira Silva *et al*., 2018). The design principles for these population types are described in Box 2. In line with what is expected of the genetic architecture of quantitative traits (Atwell *et al*., 2010), most studies find multiple QTLs for photosynthesis‐related traits with a small effect size (Yan *et al*., 2015; de Oliveira Silva *et al*., 2018), which confirms the highly polygenic nature of the trait. Nevertheless, large‐effect QTLs were found by Oakley *et al*. (2018) who assessed photosynthetic performance in a large RIL population of Arabidopsis exposed to cold stress. They concluded that the recovery of photosynthesis to cold was controlled by only a few loci, which indicates that some specific photosynthesis‐related traits are influenced by functional genetic variation in a limited number of genes. Adachi *et al*. (2011) characterised several regions associated with improved leaf photosynthesis by phenotypic analysis of a backcrossed NIL population composed of a commercial rice variety (Koshihikari) containing introgressions from a cultivar with high photosynthesis (Habataki). Subsequent introgressions of two of these loci into the Koshihikari genome significantly improved both total accumulated biomass and the rate of photosynthesis of the recipient genotype (Adachi *et al*., 2014). As such, Adachi *et al*. (2011, 2014) showed that improving photosynthetic efficiency through conventional breeding is a feasible strategy for further improving rice yield.

To summarise, photosynthesis‐related traits are generally controlled by many QTLs with a small effect size and this reflects the results from genome‐wide analyses of photosynthesis‐related genes. Large‐effect QTLs can nevertheless be detected for specific photosynthesis‐related traits, which signifies the relevance for forward genetic screening. A random genomic region of a few million base pairs in size – which is a common size for introgressions in NIL populations and of QTL confidence regions identified in RIL populations – can easily encompass hundreds of genes, of which only one may be involved in the specific trait. To localise the causal gene(s) in such a gene pool would require a significant effort through follow‐up fine mapping experiments (Adachi *et al*., 2017). Given the lengthy breeding process needed to improve the photosynthesis of crop species, based on newly identified genes, and the few decades left to meet the increased global demands of plant production (Kromdijk and Long, 2016), a more efficient gene identification pipeline is desirable. An advantage of GWAS over conventional bi‐parental mapping population studies is the greatly improved genomic resolution with which causal genetic loci can be pinpointed (Box 1). As such, GWAS have the potential to result in much smaller lists of candidate genes that may control the traits of interest, and at a high density of SNP markers many associated loci may be identified. This would potentially allow faster identification and application of newly discovered genetic variation.

There have so far been only a limited number of GWAS aimed at elucidating photosynthesis‐related traits, and an overview of all published studies we could identify is given in Table 1. Where generally few highly significant QTLs can be found in bi‐parental mapping studies, most GWAS report numerous trait–marker associations. Many of these reach just above the LOD thresholds set to declare a region of significant interest, even after strong statistical corrections for population structure and/or experimental variance are applied (e.g. Hao *et al*., 2012). Most make only a small contribution to the total phenotypic variation. These findings illustrate the classic dichotomy between GWAS and bi‐parental mapping studies; the former are more accurate at pinpointing causal variants and the latter are more powerful at detecting phenotypic differences caused by such variants (Bazakos *et al*., 2017). Nevertheless, QTLs in GWAS often identify SNPs that locate in or near genes that have a predicted role in photosynthesis‐related traits following Gene Ontology enrichment analyses, which sets the precedent for a closer investigation of those genetic loci (Dhanapal *et al*., 2016; Herritt *et al*., 2016; van Rooijen *et al*., 2017; Wang *et al*., 2017). Functional characterisation of these genes may greatly advance our knowledge of the genetic regulation of photosynthesis and potentially lead to the discovery of new traits and genes that could be targets in plant breeding programmes (Box 3). Such characterisation will require additional physiological analysis of near‐isogenic genotypes, such as mutants and wild types, including detailed, low‐throughput leaf or plant gas exchange measurements, comparative transcriptomics, promoter studies, etc., as is common in gene function analysis.

Box 3 **Highlights of two studies on functional genetic variation of plant photosynthetic efficiency**

These studies have been selected to demonstrate the potential of using genetic mapping approaches to unravel genetic variation in photosynthetic traits up to the DNA sequence level. Causal genetic variation in selected candidate genes and their effects were confirmed in follow‐up reverse genetics, and as such these studies fulfil the aim of characterising the impact of genetic variation on the phenotype.

**Example 1: Natural variation in OsPsbS‐1 affects NPQ in rice**

Improving the rate at which NPQ is induced and relaxes will ensure that the level of photoprotection matches the level of incoming sunlight, which fluctuates drastically in field‐grown crops (Murchie, 2017; Ruban, 2017). This trait is increasingly being recognised as a priority target for genetic improvement to boost crop productivity (Long *et al*., 2015; Taylor and Long, 2017). Violaxanthin de‐epoxidase, zeaxanthin epoxidase and PsbS are the key regulators of the early, energy‐dependent phase of NPQ (also known as the qE phase). Kromdijk *et al*. (2016) overexpressed these genes in tobacco and observed a higher accumulation of biomass in tobacco plants growing under field conditions compared with wild‐type control plants, demonstrating the potential to improve NPQ to greatly benefit crop yield (Long *et al*., 2015).

Wang *et al*. (2017) explored natural variation of NPQ in 529 field‐grown rice accessions and made use of a handheld pulse amplitude modulation fluorometer to assess the rate of NPQ in excised leaf parts. Two major marker–trait associations were discovered, one of which located to *OsPsbS‐1* and explained more than 40% of the total genetic variance for NPQ in the rice diversity panel. Major allelic variation – which included a 2674‐bp InDel located near the promoter region of *OsPsbS‐1* – explained significant differences in the rate of NPQ, while no functional genetic variation was found that affected the strongly conserved protein sequence. Subsequent analysis confirmed that allelic regulation of *OsPsbS‐1* expression was strongly correlated with NPQ. The ability to detect significant functional variation in *OsPsbS‐1* by means of genetic mapping can be considered as a proof of concept of the idea that candidate genes belonging to important photosynthetic processes can be retrieved using genetic mapping approaches. Consistent with the hypothesis put forward by Müller *et al*. (2001) that the xanthophyll cycle and PsbS alone cannot account for total NPQ that takes place in plants during qE, Wang *et al*. (2017) discovered more than 30 additional loci that affect NPQ, some of which explained medium to high proportions of the phenotypic variance. This suggests that the regulation of NPQ in rice can be improved beyond the known core genes. Several of the marker–trait associations were confirmed using a bi‐parental F_2_ mapping population, and thus Wang *et al*. (2017) also demonstrated the power of applying both association panels and bi‐parental mapping populations to discover and validate functional allelic variation in genetically complex traits such as photosynthesis.

**Example 2: The role of YELLOW SEEDLING 1 in the high‐light acclimation response of Arabidopsis**

Plant acclimation to changes in irradiance intensity induces various responses at the genetic, molecular and physiological levels (Athanasiou *et al*., 2010; Bailey *et al*., 2004; Kouřil *et al*., 2013; van Rooijen *et al*., 2018). van Rooijen *et al*. (2015) presented the first association mapping study in which high‐throughput phenotyping of photosynthesis‐related traits was employed to elucidate these responses in Arabidopsis. They performed a GWAS of 344 accessions of Arabidopsis to study light acclimation after a stepwise increase in growth light intensity, after which photosynthetic efficiency (*F*_q_′/*F*_m_′ or φPSII) was tracked multiple times a day for 4 days. Short‐ and long‐term acclimation responses were identified, leading to the discovery of marker–trait associations that were present in different phases of light acclimation or throughout the experiment, effectively dissecting long‐term light acclimation into different phases with the potential to derive different molecular mechanisms that account for them. By employing quantitative complementation methods with knockout mutants (Turner, 2014), allelic variations of several candidate genes were investigated to determine the light acclimation response in Arabidopsis. Allelic variation in *YELLOW SEEDLING 1* (*YS1*) made a significant contribution to long‐term acclimation to high light. YS1, a pentatricopeptide repeat protein, locates to the chloroplasts and is involved in the modulation of RNA and chloroplast development (Kindgren *et al*., 2012; Zhou *et al*., 2009). It may be a key player in the development of chloroplasts in young leaves in response to high light conditions (van Rooijen *et al*., 2017). This finding may contribute to the little‐understood transcriptional regulation of photosynthesis (Imam *et al*., 2014; Yu *et al*., 2014) and it demonstrates the power of using chlorophyll fluorescence in a high‐throughput phenotyping facility to dissect a complex photosynthetic trait.

These studies are a first probe into identifying and characterising alleles of genes affecting photosynthetic processes. The extent to which these or other alleles may improve crop yield is unknown and may be tested in follow‐up experiments.

## Challenges of Phenotyping Photosynthesis‐Related Traits

**Figure 4:**
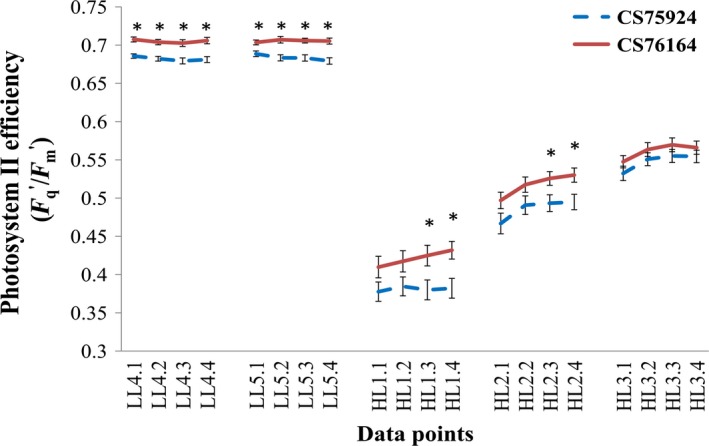
High‐throughput analysis of the response curve of natural Arabidopsis accessions Tanzania 1 (CS75924) and Landsberg *erecta* 0 (CS76164) to a stepwise increase in photosynthesis, starting from the second day of observations. Plants were grown as described by van Rooijen *et al*. (2015). Twelve replicates of each genotype have been assessed. Error bars depict ±1 standard error. Asterisks indicate significant (*P* < 0.05) differences over time and have been determined by applying Student's *t*‐test. If no asterisk is indicated at a time point there was no significant difference between the photosynthetic efficiency of the two accessions. Each set of four data points, connected by a line, represents measurements for a single day [low light (LL), 100 μmol m^−2^sec^−1^ or high light (HL), 500 μmol m^−2^sec^−1^]. Data points should be read as follows: measurement of LL1.1 is taken at 09:00, LL1.2 at 11:30, LL1.3 at 14:30 and LL1.4 at 16:00, and the same for HL.

Diversity panel and bi‐parental genetic mapping populations have been assessed for a large variety of different phenotypes related to photosynthesis. In the majority of these studies, however, single measurements are assumed to give a representative summary of the trait performance of plants. A particular problem with photosynthesis is that it quickly adapts to changes in the physical environment of the plant (e.g. to changes in light intensity, temperature and water or nutrient availability). Photosynthesis also responds strongly to stress, it often shows a diurnal rhythm and it changes according to the developmental stage of the plant. Choosing the exact timing for phenotypic evaluation of environmental responses to photosynthesis can be very challenging, as shown by the response curve of photosynthetic efficiency after a stepwise increase in light irradiance generated by high‐throughput phenotyping in Figure 4. The outcome of the statistical test drastically changes over time, and even within a single day. Thus, if only a single measurement of the plants was made its timing would determine the conclusion about whether future investigations would be worthwhile or not.

Even when applying stable light regimes that are used to reduce undesired phenotypic variation, both short‐ and long‐term genetic variation in photosynthetic performance can be detected in the appropriate phenotypic setting (Flood *et al*., 2016b). This fluidity of photosynthesis creates particular challenges for its measurement. Accurate phenotyping of photosynthesis usually requires multiple measurements over the course of the day and over the life of the plant, or at least over a period long enough to encompass the changes in the process that are relevant to the photosynthesis‐related trait of interest. In this section, various aspects of photosynthesis are discussed which require future attention in genetic mapping studies, but which impose serious challenges to phenotyping strategies. Photosynthetic responses due to environmental change are among those where most genetic variation is expected and novel traits are to be discovered (Lawson *et al.,* 2012), but which are simultaneously the most difficult to properly track (Murchie *et al*., 2018; Rungrat *et al*., 2016).

### Fluctuating light

Light is the most variable resource throughout a plant's life. Plants growing under natural conditions experience constant fluctuations in the availability of light. Apart from the diurnal sun cycle, fluctuations are caused by the waving of leaves in the upper canopy, causing shadows on lower leaves (sunflecks), and drifting clouds blocking and exposing sunlight (cloudflecks) (Kaiser *et al*., 2018). Long‐term light fluctuations include changes in weekly weather conditions and even seasonal variation, which affect plant development and photosynthetic efficiency. Recent insights into mismatches between incoming light and the ability to put that energy into use in carbon assimilation as a result of light fluctuations showed that these can cause considerable growth penalties and reduction in photosynthetic carbon assimilation in photoautotrophic organisms (Zhu *et al*., 2004; Graham *et al*., 2017; Vialet‐Chabrand *et al*., 2017; Taylor and Long, 2017; Morales *et al*., 2018; Slattery *et al*., 2018). This has been further demonstrated in Arabidopsis plants that lack functional genes involved in photoprotection and light adaptation, such as in the PsbS *npq4‐1* (Li *et al*., 2002) and *glucose 6‐phosphate*/*phosphate translocator 2* (*gpt2*) (Athanasiou *et al*., 2010) mutants. Improvement of regulatory processes and adaptation mechanisms that allow plants to improve their physiological responses to fast and slow changes in light availability is highlighted as a promising path for improvement of crop photosynthetic efficiency (Long *et al*., 2015; Kromdijk *et al*., 2016; Taylor and Long, 2017; Morales *et al*., 2018).

Plants have developed various types of adjustment mechanisms to cope with incoming light, of which the short‐term photoprotective mechanism of NPQ is one of the best investigated. In contrast to most traits related to light acclimation, rapid protocols are available for evaluating NPQ (Baker, 2008) and these have been successfully applied to discover significant allelic variation in *PsbS* in field‐grown rice (see Box 3). Beyond PsbS‐driven NPQ, additional mid‐ and long‐term adaptations include movement of chloroplasts (Bailey *et al*., 2004; Wada *et al*., 2003) and the redistribution of light‐harvesting complexes and reaction centres at the molecular level (Kouřil *et al*., 2013), as well as long‐term adaptations in leaf architecture (Tardieu *et al*., 2017; Vialet‐Chabrand *et al*., 2017). The constant switching of photosynthetic activity in response to changes in irradiance in both long and short terms requires a complex network of signalling mechanisms and regulatory gene networks in order to drive physiological adaptation; as sessile organisms, plants have to adapt physiologically as they are largely unable to evade increased irradiance or to relocate themselves to brighter spots under conditions of light shortage. Substantial heritability was found by van Rooijen *et al*. (2015) for several photosynthesis‐related traits present during acclimation to a stepwise change in light intensity for 12 natural Arabidopsis accessions. Measuring rapid plant responses, long‐term acclimation and the relationships between these traits over time will impose challenges for high‐throughput phenotyping and interpretation of short‐ and long‐term light adaptation in populations of a size that allows bi‐parental and/or association mapping.

### Crop canopy architecture

In crop canopies, light availability is unevenly spread between top leaves and shade leaves and this affects the rate of photosynthesis between different canopy levels (Wilson and Cooper, 1969; Stewart *et al*., 2003; Zhu *et al*., 2012; Murchie, 2017). Strong discrepancies between incoming irradiance at the top canopy and bottom leaves are particularly prevalent in densely grown, vertically structured crop species such as maize, soybean, wheat and other cereals. Much of the scientific attention in crop modelling is on sun‐exposed leaves, but shade leaves are a significant factor that may account for over 50% of carbon assimilation in crop canopies (Zhu *et al*., 2004, 2012). An uneven spread of incoming irradiance leads to heterogeneous exposure of plant leaves across the crop canopy and introduces patches of activated and de‐activated photoprotective mechanisms (Murchie, 2017), which leads to de‐optimisation of photosynthetic efficiency, carbon assimilation and eventually crop biomass accumulation. While optimising canopy architecture together with leaf‐level functioning in the canopy is a clear route to improving photosynthetic efficiency of the canopy, and the canopy is the agricultural unit of production, the difficulties of fully assessing canopy architecture and within‐canopy functioning are technologically burdensome. Not only must the canopy be mapped in space and time, but processes within the canopy (both physical and physiological) also need to be tracked in detail, which at the moment is not feasible on a large scale over time. Decreasing leaf chlorophyll content to allow more radiation into the canopy is a possible route to improving canopy photosynthetic efficiency (Ort *et al*., 2011, 2015). This should result in minor reductions in overall photosynthetic efficiency while significantly saving on nitrogen input (Slattery *et al*., 2018; Walker *et al*., 2018). Therefore, this trait has received considerable attention in GWAS (Dhanapal *et al*., 2016; Hao *et al*., 2012; Lin *et al*., 2017; Wang *et al*., 2015).

### Photosynthesis affected by abiotic stresses

The importance of the effect of the environment on the photosynthetic machinery is reflected by the number of genetic studies that aim to investigate plant–environment interactions (Fiedler *et al*., 2016; Herritt *et al*., 2016; Oakley *et al*., 2018; Strigens *et al*., 2013). The response of the photosynthetic machinery to cold is the most popular area of study, which may be related to the conspicuous effect that low temperatures have on crop growth, especially in cool‐temperate regions. Cold stress slows down the activity of photosynthesis – as it does all metabolic and diffusion‐dependent processes – but low temperatures slow down plant growth more than photosynthesis, which results in photosynthesis becoming restricted by sink activity. Any restriction of photosynthesis will increase the extent of either photodamage to PSII (also known as photoinhibition; see Long *et al*., 1994; Takahashi and Murata, 2008; Nishiyama and Murata, 2014) or the extent of slowly reversible down‐regulation of PSII (Demmig‐Adams and Adams, 2006; Murchie and Harbinson, 2014). *F*_v_/*F*_m_ represents the maximum quantum yield capacity of the photosynthetic machinery in plants, and can be determined on the basis of chlorophyll fluorescence. In the case of photodamage, low temperatures will simultaneously inhibit the synthesis of the D1 protein. Photodamage to PSII and the slowly reversible down‐regulation of PSII will both manifest themselves as a slowly reversible loss of the *F*_v_/*F*_m_ chlorophyll fluorescence parameter and are thus difficult to simply distinguish from each other (Murchie and Harbinson, 2014). This slow reversibility of the loss of *F*_v_/*F*_m_ results in a loss of photosynthetic efficiency under more physiologically permissive conditions, and thus in lost productivity (e.g. Stirling *et al*., 1991). Photoinhibition and slowly reversible down‐regulation of PSII are universally induced by many abiotic stressors, including, but not limited to, salinity, drought and heat stress (Nishiyama and Murata, 2014), even though there is variation in the exact physiological causes (e.g. ion toxicity, which interferes with protein cofactors, is specific to salinity stress). As photosynthesis is the main driving force behind plant productivity, the discovery of traits that maintain photosynthetic functioning under environmental pressure is highly desirable (Rungrat *et al*., 2016). *F*_v_/*F*_m_ (see Box 2) has been widely adopted in plant science to assess stress in plant leaves, primarily because unstressed leaves always tend to reach a value of around 0.83 for this trait in all plant species (Björkman and Demmig, 1987) but also because it is easily assessed using handheld devices (Murchie and Lawson, 2013). Generally, the use of the dark‐adapted *F*_v_/*F*_m_ to measure stress means that photosynthesis is being used as a probe of the plant's physiological state – the loss of photochemical efficiency may be a secondary effect to a more significant stress acting elsewhere. The loss of photosynthetic function in plants due to abiotic stresses is best described as a curvilinear transitory processes rather than a stepwise physiological transition. Insights into these plant response curves for photosynthesis may provide valuable information on how the photosynthetic machinery is preserved under environmental constraints – this is important for the recovery of photosynthetic efficiency after the stress has been alleviated. Response curves can only be constructed by repeated direct measurements of photosynthesis‐related traits and can subsequently be used to map specific phases of stress induction, recovery and adaptation.

### Photosynthesis affected by biotic stresses

Next to abiotic stresses, crop plants are being attacked continuously by different types of organisms that may have a severe impact on the integrity of plant tissue. Imaging techniques are being adopted as a convenient method to detect early infections of plant leaves (Scholes and Rolfe, 2017; Araus and Cairns, 2014; Singh *et al*., 2016; Zhao *et al*., 2016). However, as with abiotic stresses, biotic stresses also affect plant photosynthesis (Cheng *et al.,* 2016) and this may affect overall plant metabolism (e.g. root infections affect the nitrogen metabolism required for supporting photosynthesis‐related proteins) (Berger *et al*., 2007) and expression of photosynthesis genes (Bilgin *et al*., 2010). Elucidating genetic variation that controls the physiological responses of the photosynthetic apparatus under biotic stress has not been studied in any of the QTL works reported in this review and might be a topic of future investigations. The difficulty in the phenotyping of such traits resides in the spatial tracking of the impact of microbes at their sites of infection and high‐resolution phenotyping would be required to do this.

Many aspects of crop photosynthesis described here may become more important in the near future, for example because heat and drought stress are thought to affect crop production (Schauberger *et al*., 2017; Zhao *et al*., 2017) or because the traits linked to certain phenotypes are highly potent to further improve the genetic yield potential of crops (Lawson *et al*., 2012; Long *et al*., 2015; Ort *et al*., 2015). Single measurements – while they can be very important (Box 2) – may still only be able to capture a limited spectrum of traits that are involved in adapting and maintaining photosynthesis under growing conditions as described here. Thus, more physiologically complete phenotyping technologies have to be adopted to truly analyse traits and genes that may contribute to improve photosynthesis.

## Towards High‐Throughput Phenotyping to Study Natural Variation in Photosynthesis

**Figure 5:**
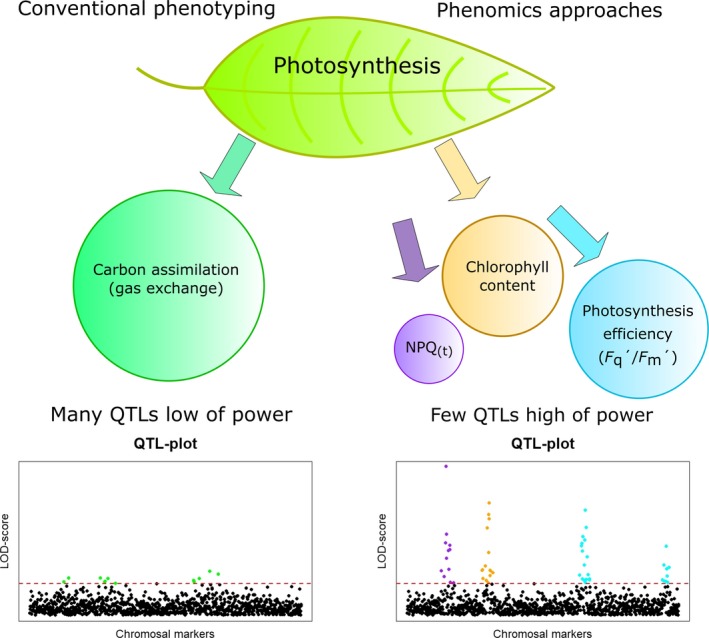
Converging genomics and phenomics. In genetic mapping studies, a heritable, quantitative trait of interest is usually determined as a one‐time assessment – here represented as an assessment of carbon assimilation in a plant leaf. Such traits are highly polygenic since different processes controlled by many more genes are responsible for their phenotypic outcomes. This results in many, barely significant (low power) quantitative trait loci (QTLs). Phenomics approaches allow the physiological and/or temporal phenotypic dissection of the trait, effectively splitting it into many traits. These traits are individually controlled by a subset of genes and/or pathways, which makes functional variation in one or a few genes belonging to this subset much more pronounced and thereby more detectable.

Phenomics is often characterised as the collection of multidimensional phenotypic data covering physiological traits, from the cellular level up to the whole organism (Houle *et al*., 2010). This includes taking into account phenotypic plasticity induced by different environments and the developmental progression of the plant (Furbank and Tester, 2011; Tardieu *et al*., 2017). In theory, the phenome of plants comprises a near endless realm of spatial and temporal phenotypes that result from the interaction of a range of plant traits with each other and the environment. In practice, plant phenomics for genetic studies is often resolved as breaking down universal, whole‐plant phenotypes into separate ones – in space or time, or both – following the logic that each ‘sub’ trait is being controlled by a smaller number of genes (Figure 5). Functional variation within such traits, which is only controlled by a subset of genes, is expected to show stronger trait associations in genetic analysis than when more general phenotypes are recorded, thus improving phenotypic resolution (Tian *et al*., 2011; Yang *et al*., 2014; Crowell *et al*., 2016; Zhang *et al*., 2017; Prado *et al*., 2018). This was conceptually verified by Crowell *et al*. (2016) who dissected the trait of panicle size into 42 distinct and targeted traits in rice. Strong marker–trait associations were found linked to distinct genes in genomic regions that were otherwise identified as weaker single loci contributing to yield, in addition to new ones that could not be detected by using a universal major phenotype. This implies that an important bottleneck in GWAS, namely its low detection power, can be overcome by high‐resolution phenotyping.

The development of imaging technology has greatly expanded in the past decade as a result of the adoption of chlorophyll fluorescence and hyperspectral techniques as sophisticated plant phenotyping analysis tools (Box 2). The introduction of handheld fluorescence devices, such as the Multispec (Kuhlgert *et al*., 2016) and tools to measure the soil plant analysis development (SPAD) index, have reduced the time needed for assessing photosynthesis‐related traits and opened the possibility for genetic mapping studies of photosynthesis even in the field. A thorough analysis of traits such as photosynthetic efficiency is still challenging, as is obvious from the limited number of phenotyping moments that can be accomplished in the time between sowing and harvesting, especially in field experiments (see Table 1). Particularly problematic is the incorporation of both short‐term physiological responses and longer‐term acclimation of photosynthesis in phenotyping assays that are highly sensitive to environmental fluctuations, that are controlled by different genetic pathways and that are acting at different time points. These features strengthen the demand for sophisticated phenotyping facilities (Flood *et al*., 2011; Kaiser *et al*., 2018; Murchie *et al*., 2018; Rungrat *et al*., 2016).

Understanding the problem of this phenotypic bottleneck led to the development of phenotyping facilities capable of high‐throughput measurement of photosynthesis‐related traits and growth parameters for a large number of plants (e.g. Cruz *et al*., 2016; Jansen *et al*., 2009). Facilities that also have the capacity to conduct GWAS or bi‐parental mapping populations are still rare, but are emerging (e.g. Cabrera‐Bosquet *et al*., 2016; Flood *et al*., 2016b; Tschiersch *et al*., 2017). A key reason for this is the financial burden of investing in photosynthesis phenotyping facilities that are able to evaluate phenotypes for hundreds to thousands of plants – corresponding to the size of diversity panels with replicates, suitable for GWAS for example – several times per day within an interval of less than an hour (Fahlgren *et al*., 2015). Considerations concerning the experimental design of such facilities have to be taken into account, and will depend on traits of interest that are to be evaluated. We have already highlighted some of the photosynthetic traits that can be evaluated, each requiring different demands with respect to measuring tools and protocols to be used by the phenotyping facility. High‐throughput data acquisition can be defined along two dimensions, temporal and spatial. High throughput on the temporal scale is achieved by repeating measurements over time. This requires a quick, but reliable, phenotyping protocol. Spatial high throughput is achieved by the ability to measure many plants within a short time, usually achieved by robotic instruments. The relevance of either axis will heavily depend on the trait under investigation, while the feasibility of doing so depends on the plant type, its developmental stage and its growing requirements.

Design approaches can be grouped into three different phenotyping principles and are generally formulated through trade‐offs between crop size, high‐throughput level of the method and the degree to which natural (field) growth conditions are met: moving the plant to the phenotyping sensors; moving the phenotyping sensors to the plant in a closed, controlled environment; and phenotyping in an open field or natural environment. The strengths and caveats of these design principles are critical to effective phenotyping.

### Moving the plant to the phenotyping sensors

**Figure 6:**
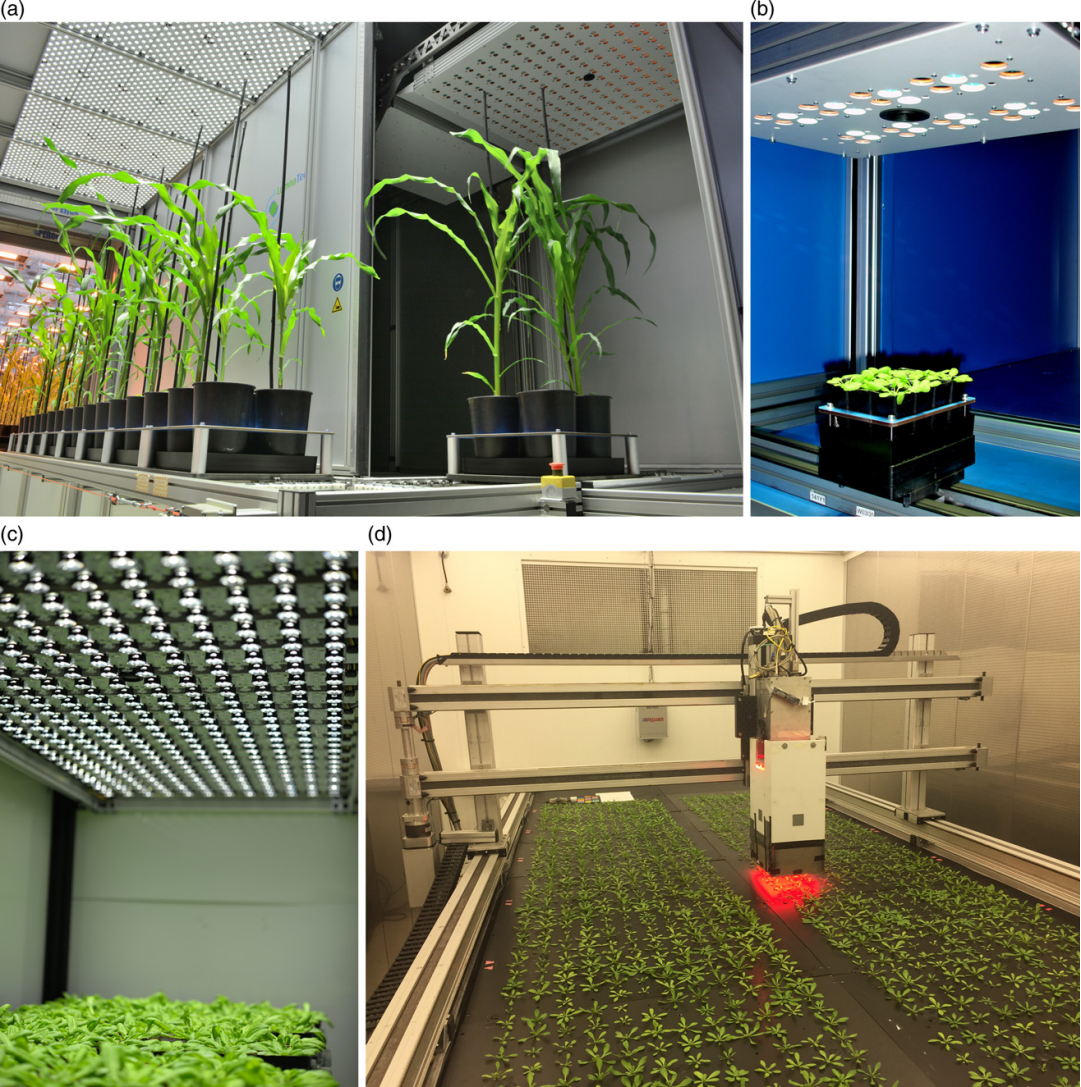
A variety of high‐throughput phenotyping facilities for photosynthesis following different experimental approaches that may be suitable for performing genetic mapping. (a) A high‐throughput phenotyping facility located at IPK Gatersleben with phenotyping sensors to measure photosynthesis‐related traits from all sides of plants the size of regular crop species (Tschiersch *et al*., 2017). (b) A close‐up of the phenotyping module for small plants at the IPK phenotyping station. (c) The Dynamic Environmental Phenoyping Imaging system developed by Cruz *et al*. (2016) at Michigan State University. (d) Phenovator, developed at Wageningen University and Research (Flood *et al*., 2016b). Photographs are used at courtesy of Henning Tschiersch, Thomas Altmann and Astrid Junker from IPK Gatersleben (a, b), Jeffry Cruz, David Hall and David M. Kramer from Michigan State University (c) and Tom Theeuwen from Wageningen University and Research (d).

The most important staple crops are rice, maize, wheat and soybean. Given their relative importance it is unsurprising that such species are of major interest in GWAS that aim to identify the genes underpinning photosynthetic efficiency (Table 1). The complex architecture of these plants, especially when compared with Arabidopsis rosettes, implies that three‐dimensional data need to be obtained for each plant from multiple phenotyping sensors, and this is difficult to do with a moving phenotyping device. One solution is to have a separate compartment with phenotyping equipment and have plants move to this by a conveyer belt system. Tschiersch *et al*. (2017) adapted a semi‐controlled high‐throughput phenotyping facility at IPK Gatersleben by incorporating chlorophyll fluorescence imaging systems into their existing phenotyping arsenal (Figure 6a,b). This is the first facility known to be able to three‐dimensionally evaluate chlorophyll fluorescence‐derived traits, such as photosynthetic efficiency, for the sizeable populations that are required for genetic analyses. ‘Moving‐the‐plant’ designs are particularly interesting for the implementation of tracking photosynthesis per leaf (Nagelmüller *et al*., 2016; Viaud *et al*., 2017), which would require sophisticated hardware for three‐dimensional imaging. Another advantage of a static phenotyping chamber is that it can be equipped with several diverse imaging devices. It can therefore potentially be used to evaluate a larger number of traits per assessment for enhanced trait integration in downstream analyses (Cabrera‐Bosquet *et al*., 2016; Tschiersch *et al*., 2017). Alternatively, modelling can be used based on parameters that are highly linked to key traits relevant to photosynthesis (Cabrera‐Bosquet *et al*., 2016). It should be noted that moving the plants may add extra variance, or noise, to the measurements, because plants will respond to movement and be exposed to differential light irradiance and angles on their way to the phenotyping sensors. The need to move plants to a single phenotyping station also limits the possibility of high‐throughput measurements of the short‐term photosynthetic responses of plants to the environment (i.e. over many minutes or hours).

### Moving the phenotyping sensors to the plant in a closed environment

Arabidopsis is an excellent model species with respect to genetic analysis of photosynthesis, because of its compact size and flat growth architecture. These properties enable efficient phenotypic platform designs that have their measuring systems positioned above the plants (Cruz *et al*., 2016; Figure 6c; Flood *et al*., 2016b; Figure 6d). Ideally, these are systems that can assess each replicate simultaneously due to the implementation of a high density of fluorescence imaging systems (Cruz *et al*., 2016). A huge advantage of such systems is that growth light can be easily manipulated without disturbing the environment of the plants, as is the case when plants are moved to the phenotyping sensors. A high density of photosynthetic phenotypic measurements over consecutive time points allows tracking of short‐ and long‐term photosynthetic responses under conditions of acclimation to a change in light conditions, as well as the short‐ and long‐term physiological acclimation responses of photosynthesis that result from abiotic stresses (Rungrat *et al*., 2016) and light fluctuation (Vialet‐Chabrand *et al*., 2017). Programmed, pre‐determined fluctuating light scenarios are of specific interest because they allow controlled experimental reproducibility, while simultaneously providing realistic phenotypic responses that are more likely to be observed in the field compared with stable light regimes (Vialet‐Chabrand *et al*., 2017). This would also allow the study of abiotic stresses such as drought under more realistic growth conditions (Rungrat *et al*., 2016). The feasibility of this approach has been demonstrated by Athanasiou *et al*. (2010) and Kromdijk *et al*. (2016), who studied differences in light acclimation regulation of control plants compared with genetically transformed plants. This yielded no physiological response when plants were grown under stable conditions, but strong differences were found in seed yield (Athanasiou *et al*., 2010) and biomass accumulation (Kromdijk *et al*., 2016) under realistic growth conditions with naturally fluctuating light. These reports are promising for translating results from climate‐controlled environments to field conditions.

### Phenomic analysis of photosynthesis in field‐grown plants

The ultimate challenge of plant phenomics is to develop high‐throughput phenotyping methods to assess plants under field conditions (Araus and Cairns, 2014; Großkinsky *et al*., 2017; Murchie *et al*., 2018). This deserves more attention, as the correlation between results from experiments in climate‐controlled conditions and those in the field is often low, which hampers the translation of results from controlled‐environment experiments to applications in agronomic conditions (Araus and Cairns, 2014). Nevertheless, phenotyping in the field will always require that many uncontrollable and irreproducible variables are taken into account, adding to the environmental variance in genetic studies which will considerably affect the power to detect QTLs for photosynthesis. This has been demonstrated by Wang *et al*. (2017), who found that NPQ was related to genetic variation in OsPsbS‐1 in only one of the experimental replications, not both, which they related to different weather conditions between years. As such, further validation of QTLs – either by using experimental repetitions or testing different environments to determine the sensitivity of the QTL to environmental fluctuations (Tardieu *et al*., 2017) – will be paramount for identifying key regulators of photosynthesis or using the QTL information to develop markers for marker‐assisted breeding, especially when testing in field conditions. Up until now there have been no automated high‐throughput phenotyping systems that can thoroughly assess plant photosynthesis in the field while accounting for in‐field light fluctuations. Overcoming such problems may probably rely on field modelling of photosynthetic performance (Murchie *et al*., 2018). Scaling down on phenotypic precision enables measuring full crop canopy structures through broad view spectral and thermal imaging, which can significantly improve high‐throughput evaluation of a variety of photosynthetic traits (Box 1). Such in‐field imaging techniques have been successfully employed by Herritt *et al*. (2016) to measure the epoxidation state of xanthophyll pigments as an indirect measure of NPQ. High‐throughput phenotyping of photosynthesis using such imaging devices should be relatively easy to include in phenotyping systems like those that support cameras which move above the crop canopy (Kirchgessner *et al*., 2016; Sun *et al*., 2018), and may provide a solution for measuring specific traits for field‐grown crops.

## Genetic Analysis of Photosynthesis Phenomics Data

### Phenomics data for GWAS

Photosynthesis phenotyping facilities, including those that can be employed in field studies, are rapidly developing to increase their trait measuring capabilities, thanks to diversifying and improved measurement protocols (Box 2). Essential for dealing with phenomics datasets will be the adoption of a streamlined processing pipeline for data handling, standardisation and storage (Großkinsky *et al*., 2015). Similar to phenomics data for other traits, photosynthesis data obtained from high‐throughput phenomics facilities will introduce new challenges associated with amalgamating gigabytes or even terabytes of imaging data and their derived measurements into a comprehensible whole with a meaningful biological interpretation (Singh *et al*., 2016; Tardieu *et al*., 2017). Latent factors which might affect plant features without being explicitly measured can be detected by applying multivariate modelling (Dormann *et al*., 2012) or training unsupervised machine learning approaches that consider the influence of hidden or unmeasurable factors (Singh *et al*., 2016). These factors can then be corrected for or integrated in biological models. Furthermore, machine learning approaches and data modelling may significantly aid the use of phenomics data in helping to identify patterns related to specific traits (Cabrera‐Bosquet *et al*., 2016; Murchie *et al*., 2018; Singh *et al*., 2016) – hereby referring to traits associated with development over time, but also traits that may be spatially linked, for example within the same leaf. This is especially useful in discerning true outliers from biologically interesting anomalies that may be lost in simpler models (Xu *et al*., 2015). To reduce data dimensions, alternative methods can also be explored, such as multitrait GWAS. Here, multivariate analyses are being employed to simplify the dimensions of phenotypic assessments which can improve the strength of QTLs – especially when different traits are assessed that share a common underlying genetic regulation (Thoen *et al*., 2016). However, care must be taken not to oversimplify data obtained from phenomics, as this could potentially hide important nuances in the data. The QTL leading to the discovery of the effect of natural genetic variation in *YS1* on acclimation of Arabidopsis to high light would not have been detected if such methods alone were employed (van Rooijen *et al*., 2017).

### From candidate gene to improving crop productivity

The ultimate goal of improving photosynthetic efficiency in plants will eventually reside in improvement of harvestable plant products (Kromdijk and Long, 2016). Improving photosynthesis, though it might result in increased total biomass accumulation, does not necessarily directly translate into greater agricultural yield. For example, Adachi *et al*. (2014) introgressed QTLs supporting carbon assimilation into an existing rice cultivar; this resulted in an increase in total biomass but not grain yield. While transgenic approaches have been successful in improving plant yield through improved photosynthesis (e.g. Driever *et al*., 2017; Kromdijk *et al*., 2016), and similar results might be achieved based on spontaneous mutants, the natural allelic variation for many photosynthesis‐related traits may be more subtle, often with many small‐effect QTLs contributing to the trait (van Rooijen *et al*., 2017). This will complicate the identification of causal genes and limit the direct use of identified alleles in breeding programmes, although it will increase our knowledge about which genes are relevant for improving photosynthesis (Box 3) – which can then potentially be used in transgenic approaches for crop improvement and inspire the search for additional allelic variants with strong affects. When many small‐effect QTLs are involved, a genomic selection approach may be adopted to improve photosynthesis, which may be more effective than breeding for a few selected loci (Hamblin *et al*., 2011). As indicated above, phenomics facilities are generally equipped with phenotyping sensors that measure growth and development traits beyond photosynthesis‐related traits, allowing the setting up of a complete, physiologically comprehensible network analysis of how all traits impact upon, or are beneficial to, crop yield. The further use of low‐throughput, but highly accurate, measuring tools, such as gas‐exchange measurement devices on contrasting genotypes identified through high‐throughput imaging analysis, will be needed to properly assess the impact of photosynthesis on crop physiology.

### Epistasis in highly polygenic traits

Due to the existence of complex regulatory networks that affect photosynthesis (Imam *et al*., 2014; Yu *et al*., 2014) and the large number of genes that are involved, QTL mapping models that solely assume additivity are likely not sufficient to derive a complete comprehension of the genetic architecture of photosynthesis. Epistasis, meaning interaction between genes resulting in non‐additivity of QTLs, has often been regarded as a rather elusive and difficult phenomenon to study, and is therefore often disregarded by plant geneticists and breeders. However, epistasis is expected to be common in highly polygenic traits (Mackay, 2014). The occurrence of epistasis is a probable explanation for the failure to detect QTLs in bi‐parental mapping approaches and GWAS, despite the presence of high heritabilities (Brachi *et al*., 2011; Lachowiec *et al*., 2015; Zuk *et al*., 2012). Early algorithms that were able to detect epistasis could only make use of small datasets and were too cumbersome to analyse the many gene‐by‐gene interactions to be found in diversity panels. New efficient ones are being developed that will make such analyses accessible to research groups without sophisticated high‐performance computing infrastructure (Ning *et al*., 2018; Tsai *et al*., 2017; Zhu and Fang, 2018), which should lead to a more complete comprehension of highly complex traits such as photosynthesis.

## Conclusion

The need for understanding and genetic improvement of plant photosynthesis is widely recognised as a vital development to combat looming future yield gap deficiencies (Flood *et al*., 2011; Lawson *et al*., 2012; Long *et al*., 2015; Zhu *et al*., 2010). This recognition, together with the development of chlorophyll fluorescence technologies that allow large numbers of plants to be evaluated within a short timeframe, has led to an increasing number of forward genetic studies aimed at detecting the genetic loci that contribute towards improving photosynthetic efficiency of crop plants. The introduction of GWAS has accelerated the speed with which candidate genes can be identified in photosynthesis‐related traits when compared with bi‐parental mapping population studies, but combining these population types to elucidate functional genetic variation and traits is likely to be the most rewarding approach (Bazakos *et al*., 2017).

In this review, we conclude that the current implementation of high‐throughput phenotyping will become necessary to elucidate the genetic architecture of photosynthesis‐related traits in mapping populations – especially with regard to the responses of photosynthesis to abiotic stresses, plant development and light acclimation. The successful identification of target QTLs and underlying genes using conventional methods greatly depends on the specific trait of interest, but has been well‐demonstrated (Box 3). The past 5 years have seen a marked improvement of and increase in the application of high‐throughput phenotyping tools, many of which can be incorporated with, employed or adapted to include sensors that measure photosynthesis. Exploring traits that contribute towards dynamic acclimation under conditions of fluctuating light may become a tractable option within a couple of years, given that more time‐efficient tools are gradually being developed. The final, and very challenging, step would be to obtain field data at a desired level of phenotypic precision comparable to what can be achieved in climate‐controlled growth facilities. The integration of both controlled and field studies will be necessary to validate gene candidacy and evaluate its application potential. We expect that the developments described here will enable further discoveries of photosynthetic trait in both science and industry to meet the ever growing demand for food for human consumption in the next three decades and beyond.

## Conflict of interest

The authors are not aware of any conflict of interest arising from drafting this manuscript.
